# COVID Vaccine Rollout for Older People: East Meets West

**DOI:** 10.1093/geroni/igab046.711

**Published:** 2021-12-17

**Authors:** Nengliang Yao, Tom Cornwell, Cheryl Camillo

## Abstract

Older adults should be one of the first groups to receive COVID-19 vaccines, because the risk of dying from COVID-19 increases with age. However, it takes time to distribute the vaccines to different countries, and the challenges in administering vaccines may differ by health system characteristics and local culture. This international symposium will discuss the vaccine rollout issues in eight countries (Isreal, Japan, South Korea, China, France, United Kingdom, Canada, and United States). We will use an interview and dialog format, instead of presentations. We will cover extensive topics including: Availability - What vaccines? Access, Acceptance, Caregivers – How are providers responding/handling caregivers wanting to be vaccinated?Cost/Financing Issues, Distribution Logistics/Transport/Safety, Lessons Learned, Mutations/Variants, Partnerships needed to vaccinate homebound patients (community partners; home health agencies, etc.), Who can/should provide vaccination? The situation with COVID-19 is still very fluid. Countries are at different stages of vaccinating older people. The chair didn't ask the speakers to write an abstract now, instead, the speakers will collect more information during the next few months and plan to have a prep meeting one month before the Annual Meeting.

